# Conservative endovascular and hysteroscopic management of a cesarean scar pregnancy in a woman with previous history of uterine artery embolization for intractable hemorrhage after cesarean section for placenta previa

**DOI:** 10.1002/ccr3.2031

**Published:** 2019-02-06

**Authors:** Kazuhisa Kitami, Wataru Koike, Hiromi Nakamura, Akihiro Takeda

**Affiliations:** ^1^ Department of Obstetrics and Gynecology Gifu Prefectural Tajimi Hospital Tajimi, Gifu Japan; ^2^ Department of Diagnostic Radiology Gifu Prefectural Tajimi Hospital Tajimi Gifu Japan

**Keywords:** cesarean scar pregnancy, computerized tomographic angiography, hysteroscopic resection, magnetic resonance imaging, transcatheter artery chemoembolization

## Abstract

Transcatheter arterial chemoembolization (TACE), followed by hysteroscopic resection of the gestational products, could be a feasible option for the conservative management of cesarean scar pregnancy (CSP) in a woman with a previous history of uterine artery embolization (UAE) with coils for intractable hemorrhage after cesarean section.

## 1 INTRODUCTION

Cesarean scar pregnancy (CSP) is a rare form of ectopic gestation, characterized by implantation of the embryo in the myometrial defects of a previous cesarean section scar.^1^ It has been postulated that CSP can manifest as two distinct subtypes: an endogenic type with progression to the cervicoisthmic space and an exogenic type with deep invasion into a scar defect with progression toward the uterine serosa.^1^

Due to a potential risk of uterine rupture and severe hemorrhage, life‐saving hysterectomy has been the mainstay of management for CSP.^1^ With recent advances in the diagnostic and therapeutic modalities, an early diagnosis of CSP provides a number of conservative management options.^1^, ^2^, ^3^ These include excision of the gestational sac by laparotomy or laparoscopy, uterine artery embolization (UAE), medical cytotoxic treatment, hysteroscopic resection, dilatation and curettage, and a combined approach of these procedures.^1^, ^2^, ^3^

However, a clear consensus on the management of CSP has not yet been established^1^, ^2^, ^3^ and different therapeutic strategies might have to be individually adopted, based on the type of CSP as determined by diagnostic imaging, and viability of the villous tissue based on the serum β‐human chorionic gonadotropin (hCG) level.^2^, ^3^

We report here, a successful conservative endovascular and subsequent hysteroscopic management of the unusual case of an endogenic subtype of CSP, with a previous history of UAE for intractable hemorrhage after a cesarean section for placenta previa.

## 2 CASE REPORT

A 33‐year‐old (gravida 7 para 3) woman was referred for suspected abnormal placentation at an estimated 7 weeks of gestation. Her obstetrical history was significant with one normal vaginal delivery, followed by two subsequent cesarean sections. Nine years earlier, the first cesarean section was uneventfully performed by a transverse incision of the lower uterine segment at another clinic, due to vulvar herpes simplex infection.

**Figure 1:**
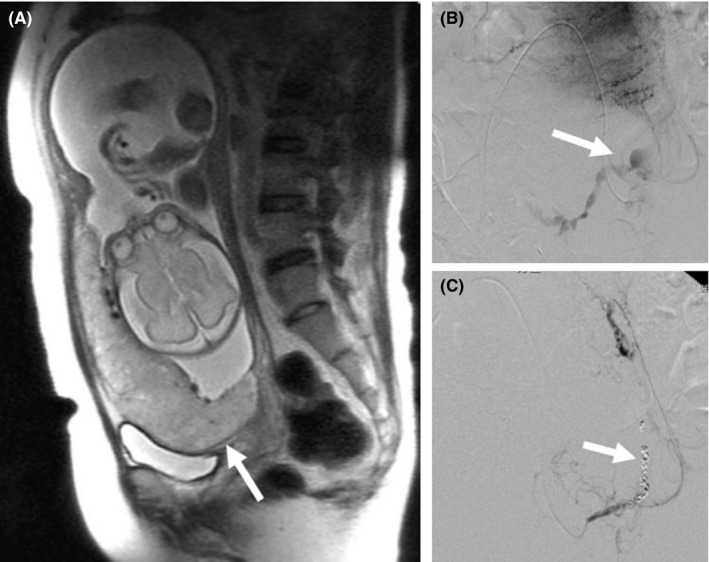
A, Sagittal T2‐weighted magnetic resonance image showing placenta previa at 28 wk of gestation. B, Pelvic angiogram showing extravasation from the left uterine artery (arrow) at postpartum hemorrhage after elective cesarean section for placenta previa at 36 wk of gestation. C, Platinum microcoil embolization of the left uterine artery (arrow) to achieve hemostasis after incomplete embolization by gelatin sponge particles

Seven years ago, after referral to our hospital due to placenta previa (Figure 1A, arrow), a second elective cesarean section was performed at 36 weeks of gestation, by an anterior vertical incision under temporary endovascular balloon occlusion of the bilateral internal iliac arteries.^4^ However, after deflation of the balloon, the peripartum period was complicated by intractable uterine hemorrhage (Figure 1B, arrow). An emergency bilateral UAE was performed with gelatin sponge particles, followed by an additional placement of platinum microcoils in the left uterine artery (Figure 1C, arrow) to achieve complete hemostasis. The subsequent postpartum course was uneventful.

**Figure 2:**
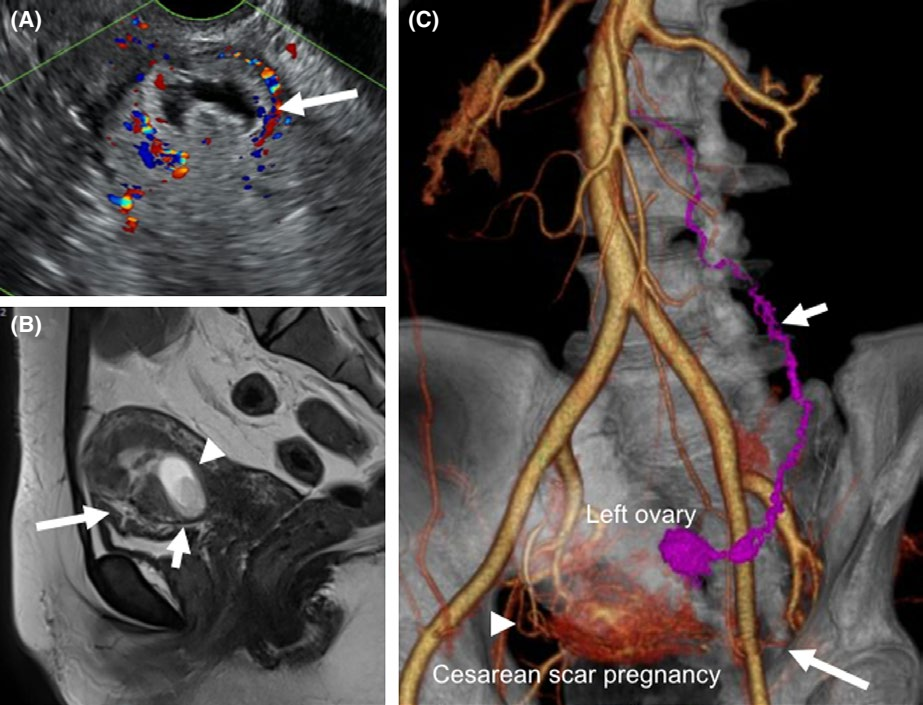
A, Transvaginal ultrasonography showing a heterogeneous mass with perivascular flow in the cesarean section scar (arrow). B, Sagittal T2‐weighted magnetic resonance imaging showing gestational products (arrowhead) implanted in the initial transverse cesarean section scar in the lower uterine segment (short arrow). Vertical scar of the second cesarean section was also noted in the anterior uterine wall (long arrow). C, Three‐dimensional computerized tomographic angiography showing the gestational products receiving aberrant blood supply from the right uterine artery (arrowhead) and anastomosing left ovarian artery (short arrow). In addition, although the main trunk of the left uterine artery was permanently occluded by the platinum coils, some persistent microvascular feeding branches of the left uterine artery (long arrow) were also recognized

At initial examination during the current referral, the transvaginal ultrasonography showed a heterogeneous mass with perivascular flow in the cesarean section scar (Figure 2A, arrow). Magnetic resonance imaging indicated an endogenic growth of the gestational products measuring 33 × 15 mm (Figure 2B, arrowhead) embedded in the transverse scar of the first cesarean section (Figure 2B, short arrow), toward the lower uterine segment. The vertical scar of the second cesarean section was also seen in the anterior uterine wall (Figure 2B, long arrow).

**Figure 3:**
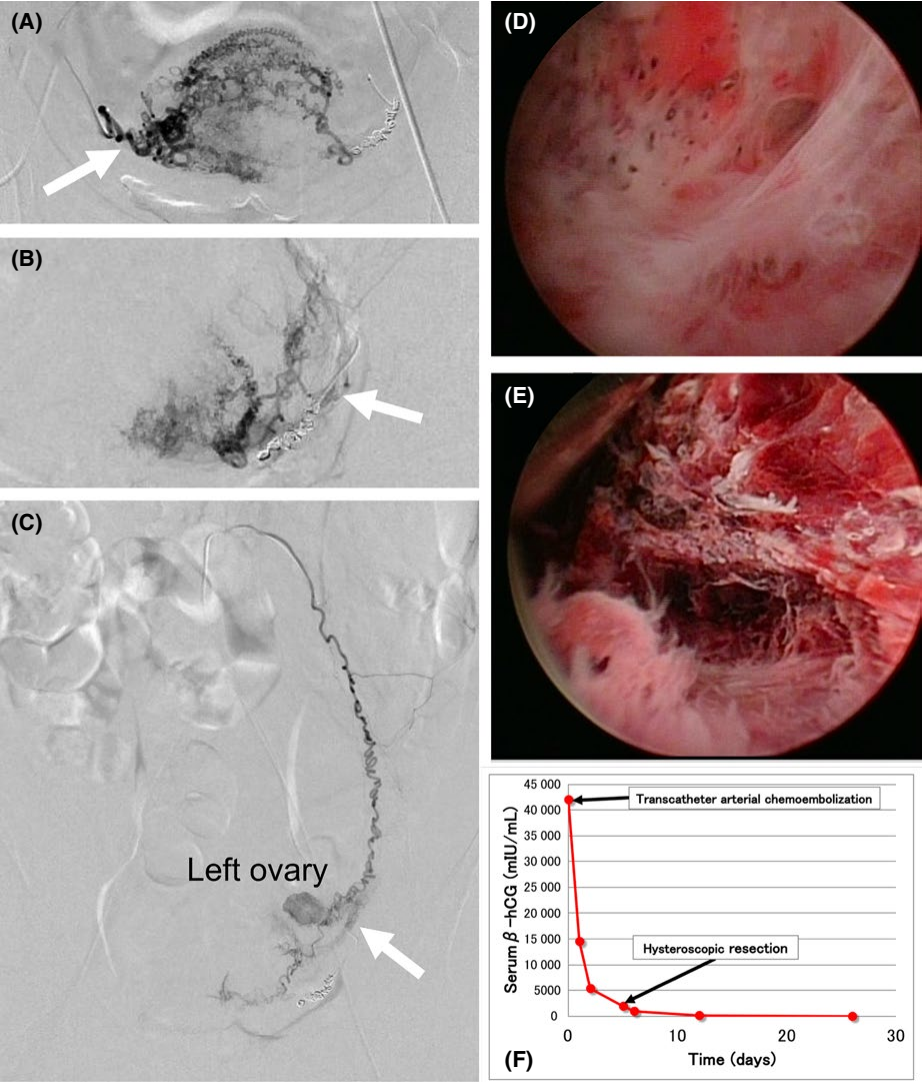
A, Digital subtraction angiography showing hypervascular staining of the blood supply from the right uterine artery (arrow). B, Digital subtraction angiography showing hypervascular staining of the blood supply from the left uterine artery (arrow). C, Persistent vascular flow from the left ovarian artery (arrow) after transcatheter arterial chemoembolization of bilateral uterine arteries. D, Hysteroscopic view showing extensive intrauterine adhesions. E, Hysteroscopic resection of the gestational products implanted in the scar of previous cesarean section. F, Changes in serum β‐hCG levels related to the treatment. After TACE followed by hysteroscopic resection, a marked decrease in the serum β‐hCG was noted. Twenty‐five days after TACE, the serum β‐hCG level returned to below the level of nonpregnant women (<6 mIU/mL) without any notable complications

A three‐dimensional computerized tomographic angiography (Figure 2C) showed the gestational products receiving blood supply from the right uterine artery (Figure 2C, arrowhead) and an aberrant anastomosing left ovarian artery (Figure 2C, short arrow). Although the main trunk of the left uterine artery was permanently occluded by the platinum microcoils, some persistent microvascular feeding branches of the left uterine artery (Figure 2C, long arrow) were also recognized. The serum β‐human chorionic gonadotropin (hCG) level was 42 022 mIU/mL (Figure 3F).

With the diagnosis of endogenic cesarean scar pregnancy established, the treatment options were discussed with the patient and her husband. The proposed management options included transcatheter arterial chemoembolization (TACE) with or without subsequent hysteroscopic resection of the gestational products^3^ and abdominal hysterectomy. An initial angiographic intervention was chosen based on the patient's strong desire to avoid laparotomy and preserve the uterus.

Digital subtraction angiography was performed as previously described.^5^ Seldinger puncture of the right femoral artery was performed for arterial access under local anesthesia. Under digital subtraction angiographic guidance, the feeding branches of the right (Figure 3A, arrow) and left (Figure 3B, arrow) uterine arteries were super selectively catheterized. For TACE,^3^ 500 μg of dactinomycin (Cosmegen; Merck & Co., Inc, Whitehouse Station, NJ) was dissolved in 60 mL of physiological saline. Half of this solution was continuously infused into the right uterine artery and the other half into the left uterine artery, each for 30 min with a dose‐controllable syringe pump. Gelatin sponge particles (Serescue; Nippon Kayaku, Tokyo, Japan) were then directly injected into the feeding branches of the bilateral uterine arteries to induce thrombosis.

However, despite a successful bilateral UAE, aberrant vascular flow to the CSP from the left ovarian artery persisted (Figure 3C, arrow). Hence, the left ovarian artery was embolized with *N*‐butyl‐2‐cyanoacrylate (Histoacryl; Braun, Melsungen, Germany) mixed with iodized oil (Lipiodol; Guerbet Japan, Tokyo, Japan). Finally, a pelvic angiogram was obtained to confirm the absence of any other feeding arteries.

After TACE, the serum β‐hCG level rapidly decreased (Figure 3F) and devascularization around the gestational products was confirmed by transvaginal ultrasonography (data, not shown). Subsequently, a hysteroscopic resection of the gestational products was attempted under spinal anesthesia.^2^ On hysteroscopy, extensive intrauterine adhesions were seen (Figure 3D). After hysteroscopic adhesiolysis, the gestational products (Figure 3E) were successfully resected.

Thedays after TACE, the serum β‐hCG level returned to below the level of nonpregnant women (<6 mIU/mL; Figure 3F), and menstruation resumed spontaneously. The patient was administered low‐dose contraceptive pills as desired by her.

## 3 DISCUSSION

With a recent increase in the rate of the cesarean deliveries, there has been an increased focus on the complications seen in subsequent pregnancies. Ranking among the rarest forms of ectopic gestation, ectopic pregnancy in a cesarean section scar is one such serious complication.^1^

Due to limited knowledge about the natural history of CSP, the process of development and pathophysiology of CSP remains to be elucidated. The potential mechanism that can explain scar implantation is that the conceptus might penetrate the myometrium through a microscopic dehiscent tract of the cesarean section scar and grow there.^1^

Although it is debatable whether the risk of CSP is related to the number of previous cesarean sections, tiny dehiscent tracts or minute wedge defects might develop from impaired healing of the cesarean incision.^1^

Furthermore, the trauma from any other uterine surgery, for example, curettage, myomectomy, metroplasty, hysteroscopy, and manual removal of placenta, might also increase the potential risk of CSP,^2^ due to persistence of damage to the endometrial tissue caused by such procedures, and affect the implantation of the fertilized embryo in the endometrial cavity.^1^

In the present case, extensive intrauterine adhesions were seen on hysteroscopy. This could be due to the damage to the endometrial tissue from the previous cesarean section for placenta previa, which necessitated UAE. As a result, the fertilized ovum, which could not achieve intrauterine implantation, might have migrated and settled in the cesarean section scar in the lower uterine segment and was growing there.

For the diagnosis,^1^, ^2^, ^3^ in addition to the clinical symptoms and serum β‐hCG levels, assessing the uteroplacental neovascularization around the CSP by using color Doppler ultrasonographic imaging is important for an initial evaluation. Subsequently, magnetic resonance imaging can accurately localize the gestational products to enable classification of the subtypes of CSP like endogenic CSP in the present case.^2^

Furthermore, the currently evolving three‐dimensional computerized tomography is extremely important when considering endovascular management, because of its accuracy and rapidity in localizing a vascular mass with its feeding vessels.^2^, ^3^ Moreover, in the present case since feeding arteries to the CSP had markedly changed due to the previous microcoil embolization of the left uterine artery, useful information about the vessels to be targeted during the interventional approach was obtained.

Cesarean scar pregnancy remains a clinical challenge for treating physicians, especially when the objective is preservation of the uterus.^1^, ^2^, ^3^ UAE is known to be an effective intervention to intercept the blood flow to the ectopic placental tissue implanted at the site of the cesarean section scar.^1^ However, if necrosis of the placental villous tissue does not occur solely by arterial embolization, complex vascular networks in the pelvic cavity might lead to revascularization of the retained placental tissue through either recanalization of the feeding vessels or collateral neovascularization, even after successful embolization.^2^ As a result, treatment failure might lead to secondary hemorrhage that in extreme cases eventually requires a hysterectomy.

Transcatheter arterial chemoembolization is a combination of intra‐arterial infusion chemotherapy and subsequent transcatheter arterial embolization.^2^ In addition to its effectiveness for localized gestational trophoblastic disease, our previous report indicated that TACE is a useful conservative measure for the management of CSP. TACE locally increases the effective dose of chemotherapeutic agents in the placental tissue with a reduction in systemic cytotoxicity, followed by interception of the blood flow to the placental tissue by arterial embolization.^2^

In the present case, because of the unusual obstetric history of UAE for postpartum hemorrhage, the major blood supply was from the right uterine artery and aberrant left ovarian artery. Therefore, left ovarian artery embolization was necessary to intercept completely, the blood supply to the CSP in addition to the bilateral UAE.

Ovarian artery embolization is known to be an effective measure to treat uterine myomas, with negligible damage to the ovarian function.^6^ In the present case, the early postintervention course was uneventful, without any signs of ovarian necrosis. Since immediate administration of the low‐dose contraceptive pill made it difficult to evaluate follicular development in the left ovary, further careful follow‐up would be necessary.

In conclusion, the present results emphasize that TACE, followed by hysteroscopic resection of the gestational products, could be a feasible option for the conservative management of CSP in a woman with a previous history of UAE for intractable hemorrhage after cesarean section.

## CONFLICT OF INTEREST

None declared.

## AUTHOR CONTRIBUTION

KK: contributed as primary manuscript author. WK: managed patient by arterial embolization. HN: managed patient. AT: heavily involved in manuscript editing.

## References

[1] Gonzalez N , Tulandi T . Cesarean scar pregnancy: a systematic review. J Minim Invasive Gynecol. 2017;24:731‐738. 28268103 10.1016/j.jmig.2017.02.020

[2] Takeda A , Koyama K , Imoto S , Mori M , Nakano T , Nakamura H . Diagnostic multimodal imaging and therapeutic transcatheter arterial chemoembolization for conservative management of hemorrhagic cesarean scar pregnancy. Eur J Obstet Gynecol Reprod Biol. 2010;152:152‐156. 20646824 10.1016/j.ejogrb.2010.05.032

[3] Takeda A , Imoto S , Sakai K , Nakamura H . Three‐dimensional computed tomographic angiography in the diagnosis and conservative management of cesarean scar pregnancy with prominent neovascularization. Taiwan J Obstet Gynecol. 2014;53:385‐388. 25286796 10.1016/j.tjog.2013.11.005

[4] Takeda A , Koyama K , Imoto S , Mori M , Sakai K , Nakamura H . Temporary endovascular balloon occlusion of the bilateral internal iliac arteries for control of hemorrhage during laparoscopic‐assisted myomectomy in a nulligravida with a large cervical myoma. Fertil Steril. 2009;91:935. e5‐9. 10.1016/j.fertnstert.2008.09.040 18990372

[5] Takeda A , Koike W , Imoto S , Nakamura H . Three‐dimensional computerized tomographic angiography for diagnosis and management of intractable postpartum hemorrhage. Eur J Obstet Gynecol Reprod Biol. 2014;176:104‐111. 24630300 10.1016/j.ejogrb.2014.02.026

[6] Hu NN , Kaw D , McCullough MF , Nsouli‐Maktabi H , Spies JB . Menopause and menopausal symptoms after ovarian artery embolization: a comparison with uterine artery embolization controls. J Vasc Interv Radiol. 2011;22:710‐715. 21514524 10.1016/j.jvir.2011.01.441

